# Biosynthesis, Characterization, and Biomedical Applications of Gold Nanoparticles with *Cucurbita moschata* Duchesne Ex Poiret Peel Aqueous Extracts

**DOI:** 10.3390/molecules29050923

**Published:** 2024-02-20

**Authors:** Uğur Kaval, Hülya Hoşgören

**Affiliations:** Department of Biology, Faculty of Sciences, Dicle University, Diyarbakır 21280, Türkiye; ugurkaval1721@gmail.com

**Keywords:** green synthesis, gold nanoparticles, *Cucurbita moschata*, antimicrobial, cytotoxic activity

## Abstract

In this study, AuNPs were biosynthesized from *Cucurbita moschata* fruit peel extracts. Biosynthesized AuNPs exhibited maximum absorbance at a 555 nm wavelength, and XRD analysis indicated that the CM-AuNPs had a particle size of less than 100 nm and a cubic crystal structure. TEM scans revealed that the gold particles exhibited a spherical morphology, with an average size of 18.10 nm. FTIR analysis revealed strong peaks indicating the presence of functional groups involved in the reduction reactions. The surface charge of the biosynthesized AuNPs was determined to be −19.7 mV. The antibacterial and antifungal activities of AuNPs against pathogen strains were assessed by the minimum inhibitory concentration (MIC) method. The cytotoxic effects of CM-AuNPs on cancer cell lines (Sk-Ov-3, CaCo_2_, and A549) and healthy cell lines (HUVEC) were investigated using the MTT method. The findings indicated that AuNPs biosynthesized by the green synthesis method using *C. moschata* peel aqueous extract had high inhibition on the growth of pathogenic microorganisms and effective cytotoxic activity against cancerous cell lines at low doses. As a result, it can be concluded that CM-AuNPs will be eminently effective in the production of antibacterial and/or anticancer drugs in the pharmaceutical, food, and cosmetic industries.

## 1. Introduction

Nanotechnology has seen a remarkable expansion in the use of nanoscale materials for various scientific and technological applications, covering a wide range of disciplines. High material properties and transport capability give increasing importance to the synthesis and applications of nanoparticles. Nanoparticles (NPs) are high-performance structures with various biological advantages [1,2,3]. Due to their small size and high volume ratio, NPs have distinct physical and chemical properties compared to macroforms [4]. AuNPs have high potential due to their biocompatibility, optical, catalytic, antimicrobial, and cytotoxic properties [5,6]. Chemical and physical methods are beneficial for the synthesis of metal nanoparticles but have disadvantages such as increased costs, toxic release, and long-term syntheses and purification [7,8]. Using biological processes for the green synthesis of NPs is a more effective technique and achieves a greater yield than other methods. Plants containing stabilizing and reducing biochemical components are used in the synthesis of green NPs [9]. According to research, the cytotoxic properties of plant-based synthesized AuNPs can effectively combat infection and cancer cell lines at different doses [10]. Flavonoids, carboxylic acids, terpenoids, quinones, ketones, aldehydes, and amides are essential phytochemicals responsible for bioreduction in plant-derived biosynthesis [11].

The material for this study, *C. moschata*, has been found to have cytotoxic effects and antimicrobial–antioxidant properties against some cancer cells [12,13]. Some studies on the characterization, bioreduction content, and biomedical uses of AuNPs synthesized by species of the Cucurbitaceae family have shown antibacterial [14,15,16] and anticancer effects [17,18,19,20]. 

This article highlights the potential and importance of the use of plant-derived compounds in biomedical research. It forms the basis for the discovery and development of therapeutic agents from natural sources. In this research, we carried out economic, rapid, and green biosynthesis of CM-AuNPs, obtained from ions derived from *C. moschata* extract as a bioinducing agent, HAuCl_4_ (a solution of tetrachloroauric (III) acid). The aim of this study is to characterize biosynthesized CM-AuNPs and evaluate their antibacterial and cytotoxic properties. Changes in the properties of phytosynthesized AuNPs in the future are expected to offer significant advantages in green nanoscience applications, in cancer treatment, and combating antibiotic resistance. 

## 2. Result and Discussion 

### 2.1. LC-ESI-MS/MS Analysis Results

**Figure 1:**
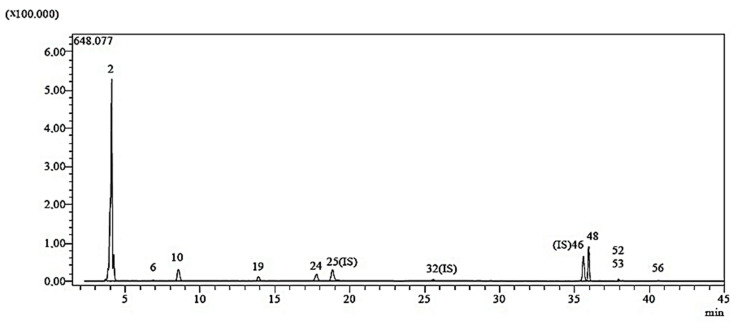
Sample chromatogram for LC-ESI-MS/MS study.

The chemical composition of *C. moschata* was investigated in this work (Table 1). Compounds detected by LC-ESI-MS/MS analysis were indicated in a standard sample chromatogram (Figure 1).

The highest concentrations of nine phenolic compounds (acacetin, kaempferol, apigenin, fumaric acid, protocatechuic aldehyde, p-coumaric acids, protocatechuic acid, naringenin and vanillin) have been found in phytochemicals (Table 1). The biological activity of CM-AuNPs can be associated with various phenolics, as demonstrated by Ahmad and Kalra [21] and Dos Santos et al. [22]. Phenolic chemicals have positive effects on human health, such as reducing the risk of several diseases like diabetes, cancer, heart disease, and neurological problems [23]. 

In previous studies, bioactive compounds in the methanol extract of *C. moschata* fruit, seed and fiber have been for therapeutic use [24]. The highest level of fumaric acid detected in this analysis is known to have antimicrobial and cytotoxic activity against certain microorganisms (*E. coli* DSM 5923 and *S. aureus* ATTC 6538) [25]. Naringenin has been known to have antibacterial and antiproliferative activity against various pathogenic bacteria in the cancer cell lines [26]. Previous studies have highlighted the inhibitory effect of apigenin against the growth of various bacteria [27]. Another study found that kaempferol inhibited the proliferation of cell lines such as HepG2, MCF-7, and A549, as well as *E. coli* and *P. aeruginosa* [28,29]. The inhibitory effect of acacetin on pneumonic diseases and on the proliferation of A549 has been demonstrated [18]. Previous research has shown that protocatechuic acid found in various plants has antibacterial activity against Gram-positive and Gram-negative bacteria, as well as fungi [30,31]. Given the simple and affordable synthesis of AuNPs, phenolic compounds (PCs) are among the most attractive conjugates for AuNPs.

### 2.2. UV-Vis Spectrum Data of CM-AuNPs

**Figure 2:**
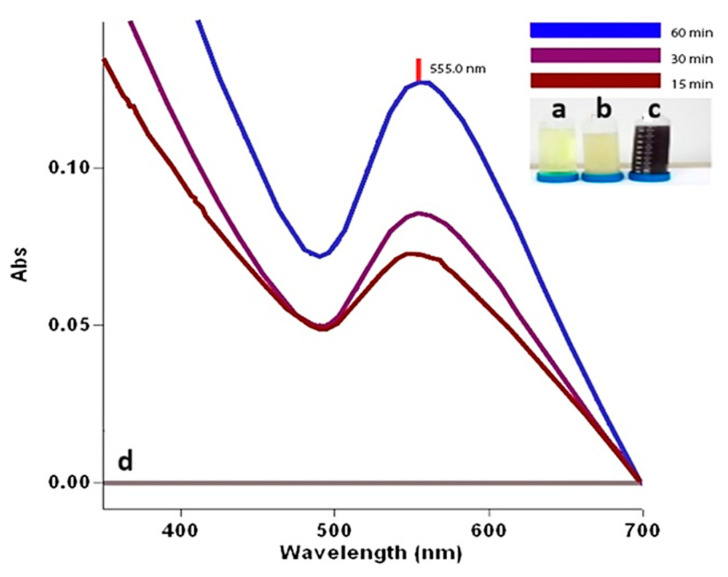
(**a**) 10 mM HAuCl_4_ solution; (**b**) the plant extract; (**c**) the color change observed is attributed to the production of AuNPs during the synthesis process; (**d**) UV-vis spectra bands showing the time-dependent formation of AuNPs.

In this study, after mixing *C. moschata* extract and 10 mM gold solution for an hour, there was an appearance of AuNPs with a prominent peak at 555.0 nm in the UV-vis spectrum (Agilent CARY 60 equipment, Agilent, Penang, Malaysia), as shown in Figure 2. A noticeable color change was observed during the green synthesis of AuNPs with *C. moschata* bark extract. The mixture has a darker appearance from a yellow to pink-red color within 20 min. The observable yellow-red color change indicating the formation and presence of AuNPs is due to the reduction of Au^+3^ to Au^0^ by biological processes. Phytochemicals in the plant extract have reduced the Au^+3^ precious metal to the Au^0^ precious form, resulting in the synthesis of CM-AuNPs. This bioreduction process showed maximum absorption bands at 555.0 nm wavelengths due to the surface plasmon resonance (SPR) phenomenon caused by vibrations on the surface of the plasma [32]. The constancy of the highest absorption value (555.0 nm) observed after color change in time-dependent samples is evidence of the synthesis of a stable structure in the formation of the reaction.

Biosynthesized AuNPs of certain species of the genus Cucurbita are compatible with the overlapping absorbance values of our study [33,34,35,36].

### 2.3. XRD Analysis Data of CM-AuNPs

**Figure 3:**
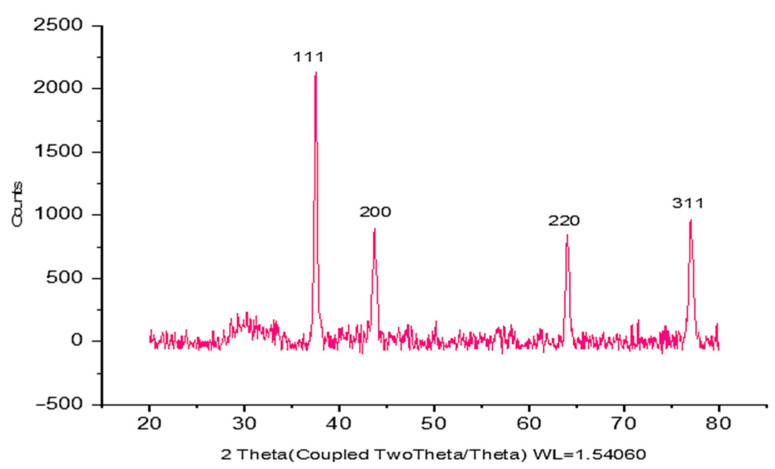
XRD spectra of CM-AuNPs synthesized by the green method.

According to the data obtained by XRD analysis, it is seen that CM-AuNPs have a cubic crystal lattice structure and 20° ≤ 2θ ≤ 80° boundary values. The crystal reflection planes of 37.768°, 43.917°, 63.687°, 76.809°, and 80.678° corresponding to 2θ values of CM-AuNPs are (111°), (200°), (220°), and (311°), respectively (Figure 3). It can be said that as the 2θ angle increases, the distance between the voids decreases; thus, a tighter bond is formed (Figure 3). In XRD analyses, the crystal size of CM-AuNPs was determined to be 24 nm using the Debye–Scherrer equation as a result of measurements taken with a D8 DISCOVER analyzer [37,38]. This result is consistent with similar findings reported in studies from the literature [39].

### 2.4. FTIR Spectroscopy Data of CM-AuNPs

**Figure 4:**
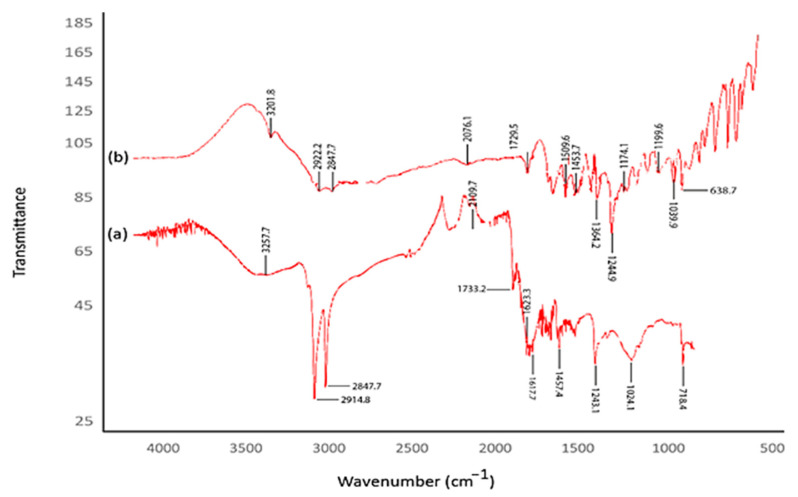
(**a**) CM-AuNP FTIR spectroscopy analysis and (**b**) FTIR measurement of *C. moschata* extract.

The FTIR spectrum was analyzed to identify the biological compounds and reducing or capping agents of the extract that may play important roles in forming AuNPs from HAuCl_4_. In this study, a Perkin Elmer Spectrum 100 brand device was used. The identified frequency variations are depicted in Figure 4. The shifts observed at 3257.7–3201.8, 2914.8–2922.2, and 28427.7–28427.7 cm^−1^ in the spectra indicate the presence of functional groups crucial for bioreduction and stability (Figure 4) [40].

When studying reducing functional groups in the formation of AuNPs, the determined peaks can be attributed to ester bonds in polyphenolic compounds, the OH-stressing vibration of phenolic compositions, and the carbonyl group’s tension vibration. To produce zero-value AuNPs, it has been shown that the biological reduction of +3 values of Au metal in an aquatic environment and its stability can be responsible for O–H, alkyne (–C=C–), and amine (–NO) groups [41,42,43,44,45].

### 2.5. SEM and EDX Profiles Analysis of Biogenic Gold Nanoparticles

**Figure 5:**
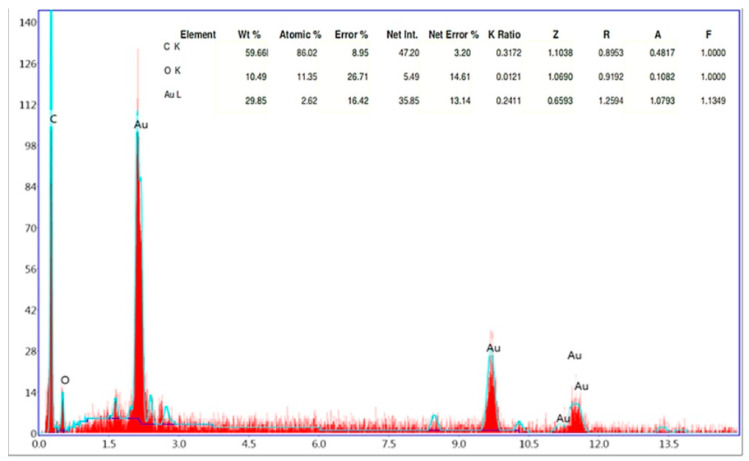
EDX analysis results of CM-AuNPs.

The EDX analysis showed that HAuCl_4_ was reduced by plant extract by strong signals of gold atoms at different energy levels. Additional plant-derived elements, such as carbon and oxygen, are identified in Figure 5.

**Figure 6:**
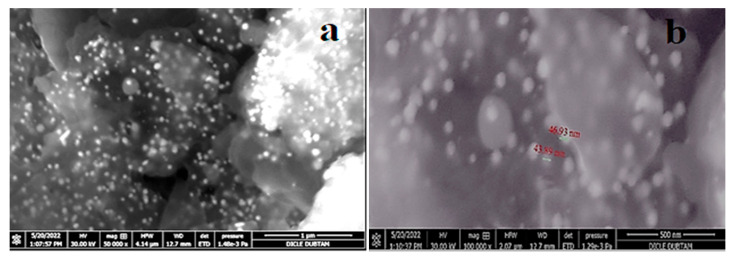
SEM analysis of synthesized AuNPs: (**a**) 50,000× and (**b**) 100,000× microscope magnification.

The EDX profile detected 29.85% of AuNPs in the synthesized elemental structure. SEM analyses conducted to determine the size and morphological properties of biosynthesized AuNPs showed homogeneous shapes of CM-AuNPs (Figure 6). SEM has demonstrated that AuNPs show a variety of morphologies, both spherical and cubic, with dimensions of less than 100 nm.

### 2.6. TEM Analysis of CM-AuNPs

**Figure 7:**
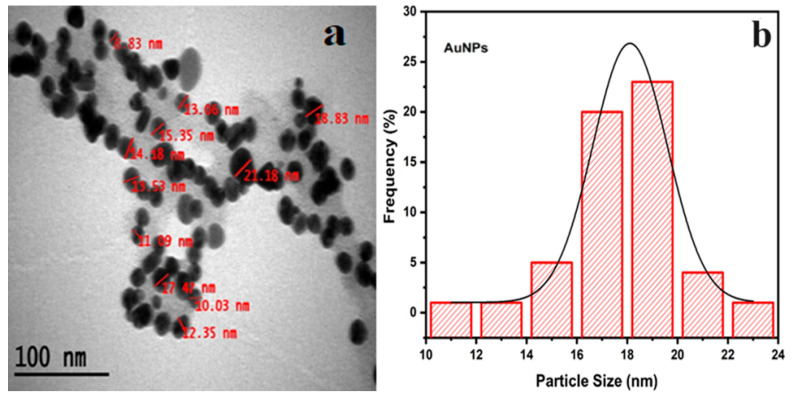
(**a**) TEM image of CM-AuNPs (100 nm). (**b**) Average size analysis of TEM analysis of CM-AuNPs.

The best images and measurements were obtained with three repeated TEM analyses. The biogenic AuNPs in Figure 7a were shown to be stable in the predominant spherical and cubic morphologies, averaging 18.10 nm in size (Figure 7b). The presence of AuNPs with a spherical morphology has been demonstrated in previous studies and has a high capacity to pass through the cell membrane (2.5 to 50 nm) [46]. In repeated measurements of AuNPs, the smallest diameter was 8.83 nm, and the largest diameter was 21.18 nm. As a result of the measurements, the standard deviation was calculated as 2.629877755 nm.

### 2.7. TGA-DTA Analysis of Biosynthesized CM-AuNPs 

**Figure 8:**
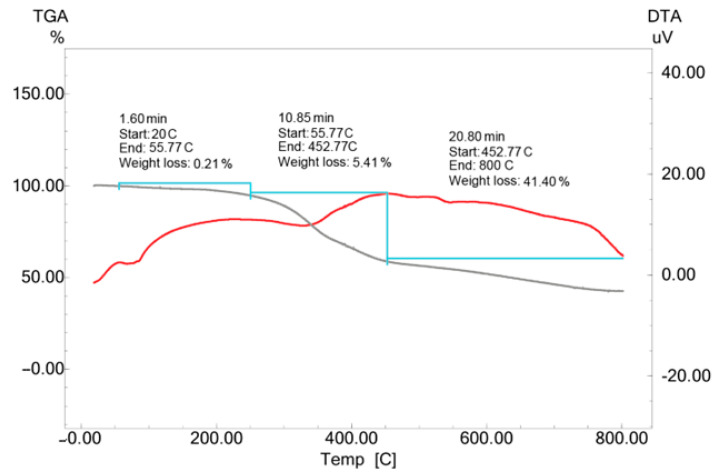
TGA-DTA results of biosynthesized CM-AuNP. Grey: mass loss, Red: Temperature increase.

The temperature resistance of CM-AuNPs synthesized with the aquatic extract of the *C. moschata* peel has been assessed in the 0–800 °C range. The TGA-DTA analysis revealed that mass loss occurred in three different temperature ranges. The first mass loss observed between 20 and 55 °C (0.21%) was associated with the evaporation of the adsorbed water. Subsequent mass losses between 55 to 452 °C (5.41%) and 452 to 800 °C (41.40%) were associated with the decomposition of bioorganic compounds (Figure 8).

In addition, a continuous loss of mass was observed up to a temperature of 800 °C, with a mass loss of approximately 41.40% at temperatures of up to 800 °C. The observed changes are thought to be caused by phytochemicals [47,48].

### 2.8. AFM of Biosynthesized CM-AuNPs

**Figure 9:**
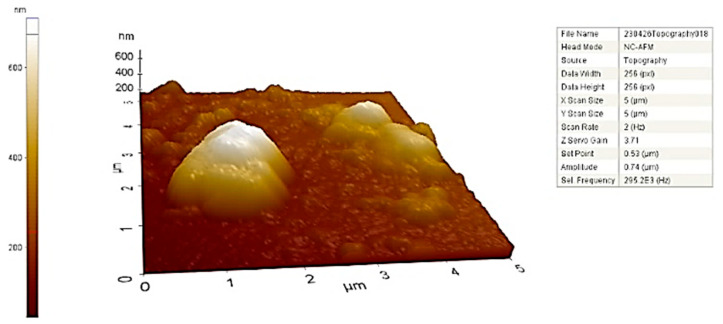
AFM micrograph topographic image of CM-AuNPs.

AFM analysis enables a more comprehensive understanding of the size and morphology of three-dimensional AuNPs. The size, shape, and surface specifications of the synthesized AuNPs have been confirmed. Figure 9 shows the topographic distribution and morphology of CM-AuNPs. The AFM micrograph showed the presence of monodispersed AuNPs with a diameter of less than 50 nm. The average particle size of biosynthesized AuNPs was shown to be between 40 and 60 nm. AFM micrographs are supported by CM-AuNPs’ TEM and SEM results, which are consistent with the literature [18].

### 2.9. Surface Charge Data of Biosynthesized CM-AuNPs by Zeta Potential Analysis

**Figure 10:**
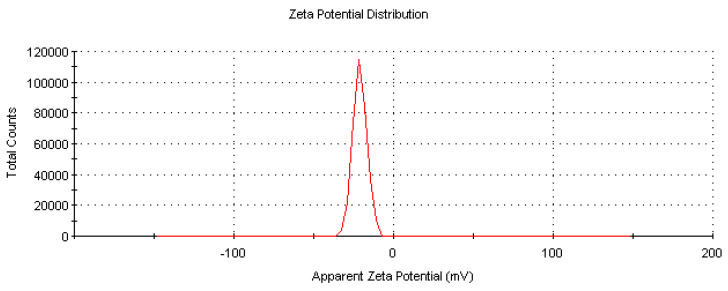
Zeta potential of CM-AuNPs.

The mobility, distribution, and stability characteristics of biosynthesized CM-AuNPs were determined by zeta potential analysis (Figure 10). As the aim of our research is to make green synthesis, the results we obtain are in sizes ranging from 10 nm to 100 nm and at levels of 0.1 mg/mL with the lowest concentration range. In the synthesis process of the nanomaterial, water was preferred as a solvent, and the optimum pH value was set at 7.8. In this study, the surface charge of the biosynthesized AuNPs was −19.7 mV. The negative value of the zeta potential indicated their physicochemical stability.

Negative-charged functional groups in the peel extract contribute to the stability of AuNPs with negative zeta potential. This negative surface charge ensures pH stability, preventing aggregation and agglomeration. Metal-based nanoparticles usually have a negative or positive surface charge, which is associated with stability. CM-AuNPs are stable and monodisperse with a negative surface charge, and their low negative charge and size distribution are important in biomedical applications. The zeta potential distribution of AuNPs synthesized by various plants may be different. For example, different values (−21.6 eV, −0.3 mV) have been found in plants of the genus Cucurbita [49,50,51,52,53].

### 2.10. Biomedical Applications of AuNPs

#### 2.10.1. Cytotoxic Effect Analysis of Biosynthesized AuNPs

Metal nanoparticles synthesized using plants are known to have antiproliferative and cytotoxic effects and significant properties against cancer [54]. The cytotoxic abilities of AuNPs are known to be inhibiting, leading to increased levels of reactive oxygen species (ROS) against cell membranes, enzymes, and cell nuclei [37]. 

This study evaluated the cytotoxic effects of CM-AuNPs on cancer cell lines using MTT analysis. To investigate potential anticancer activity, different cell lines such as HUVEC, Sk-ov-3, A549, and CaCo-2 were exposed to AuNPs at concentrations ranging from 6.25 μg/mL to 100 μg/mL. The cells that interacted with CM-AuNPs were incubated for 48 h. The cytotoxic effects of CM-AuNPs at various doses were identified, and they are shown in Table 2. The findings of this study have demonstrated that CM-AuNPs have remarkable cytotoxic effects even at low concentrations (Table 2).

**Figure 11:**
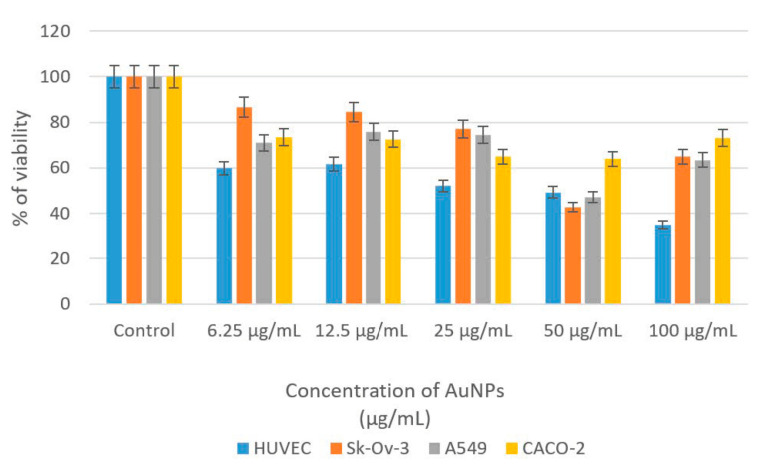
Inhibition effects of CM-AuNPs on HUVEC, Sk-Ov-3, A549, and CaCo-2 cancer cells’ % of viability rates.

This study revealed a clear association between the concentration of nanoparticles and the extent of cell growth inhibition. CM-AuNPs exhibited better antiproliferative activity with 47.19% viability in A549 and 42.76% in Sk-Ov-3 at a 50 μg/mL concentration. Various concentrations ranging from 0 (control) to 100 µg/mL were used during the experiment. For 24 h, the 50% inhibition concentration (IC_50_) was determined for HUVEC (44.6747 μg/mL), Sk-Ov-3 (114.0851 μg/mL), A549 (144.2599 μg/mL), and CaCo-2 (1871.2831 μg/mL). The decrease in cell viability relative to the administered dose indicates that AuNPs have a cytotoxic effect on the indicated cell lines (Figure 11).

Human umbilical vein endothelial cells (HUVECs) (CRL-1730) cell line, human ovarian adenocarcinoma (Sk-Ov-3) (HTB-77) cell line, human lung cancer (A549) (CRM-CCL-185) cell line, and human colon adenocarcinoma (CaCo-2) (HTB-37) cell cultures were obtained commercially from the American Type Culture Collection (ATCC) with the project number FBE.21.013. Cytotoxicity studies of the biosynthesized AuNPs were performed in the Cell Culture Laboratory of Dicle University, Faculty of Veterinary Medicine.

Table 3 shows the concentrations of AuNPs that inhibit the viability of some cancer cell lines, as determined by the green method of synthesis. 

#### 2.10.2. Evaluation of Antimicrobial Activities of CM-AuNPs

**Figure 12:**
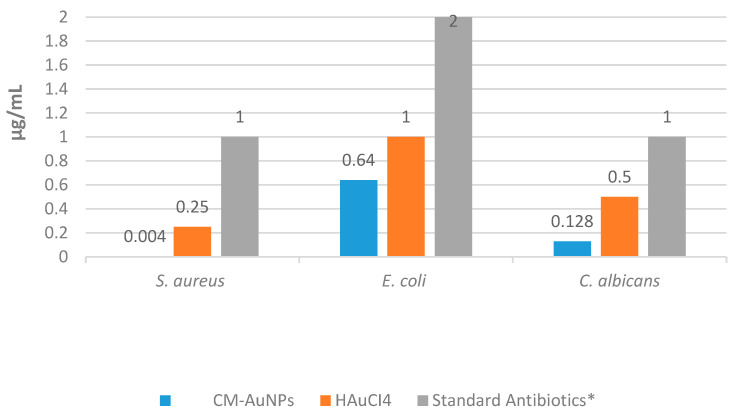
Antimicrobial MIC values of synthesized CM-AuNPs, HAuCI_4_ solution, and antibiotics.

In this study, microdilution analysis was used to determine the minimum inhibitory concentrations (MICs) of AuNPs, which are effective against *E. coli* ATCC 25922 (Gram-negative), *S. aureus* ATSC 29213 (Gram-positive), and *C. albicans*. In this study, the antimicrobial properties of AuNPs were studied by comparing them with commercial antibiotics such as fluconazole, vancomycin, and colistin. This study showed that CM-AuNPs have significant effectiveness against *S. aureus*, *E. coli*, and *C. albicans* at extremely low concentrations (0.004 μg/mL, 0.64 μg/mL, and 0.128 μg/mL) (Figure 12). The antimicrobial mechanism of biosynthesized AuNPs arises from the influence of Gram-positive and Gram-negative bacteria on the membrane’s surface structure. These AuNPs are thought to be effective in reducing adenosine triphosphate (ATP) levels, promoting oxidative stress, and inhibiting ribosomal structures [58,59]. AuNPs are thought to be effective in antimicrobial activity due to their surface load, size distribution and concentration [60]. The presence of nanoparticles increases the formation of reactive oxygen species (ROS) during interaction between pathogens and nanoparticles. The deterioration of critical biomolecular structures, such as cell membranes and nuclear membranes, is seen because of increased levels of ROS [61].

It is known that the mechanisms of antibiotic resistance of pathogenic microorganisms have been developed. However, AuNPs have shown significant potential as alternative antibacterial agents [4,40]. Biogenic AuNPs derived from species of the Cucurbitaceae family have been shown to significantly inhibit the growth of bacteria and some fungus species at various concentrations. Table 4 shows the amounts of AuNPs that effectively inhibit the growth of microorganisms in various green synthesis studies.

CM-AuNPs exhibited activity on Gram-positive and Gram-negative species at very low doses compared to the standard antibiotics tested (colistin and vancomycin). Moreover, the suppressive effect of CM-AuNPs on *C. albicans* was found to be significantly more effective at concentration than the standard antibiotic (fluconazole) (Table 5). When analyzing the doses required for inhibiting the growth of the tested pathogens, it was observed that AC-AuNPs exhibited significantly lower concentrations compared to normal antibiotics and HAuCl_4_ solution. 

## 3. Materials and Methods

### 3.1. Process of Extraction and Biosynthesis

The sample of *C. moschata* was obtained from a public market in Diyarbakır, Turkey. The pericarps, also known as pells, of *C. moschata* fruit underwent a sequential rinsing process using tap water, followed by distilled water, and were afterward dried at room temperature. The mixture, containing 250 g of dried peel and 750 mL of distilled water (1:3 ratio), was heated to 50 °C. The plant extract was then cooled to room temperature before being put into a 0.45 mm membrane filter. In the framework of the experimental research, the cooled filtrate was subsequently kept in a refrigerator set to a temperature of 4 °C.

Tetrachloroauric (III) acid (hydrogen tetrachloroaurate (III) trihydrate), ACS, 99.99% (metal-based), Au 49.0% min, CAS: 16961–25-4 was purchased from Sigma-Aldrich (St. Louis, MO, USA) and used to produce 10 millimolar (mM) of 250 mL of HAuCI_4_ solution. The 250 mL HAuCI_4_.3H_2_O solution was mixed with 750 mL of plant extract (1:3 ratio). The solution was mixed for 15 min at 50 °C using a magnetic heater at a rotation rate of 750 rpm. Finally, the mixture was allowed to cool at room temperature. The mixture was observed at intervals of 15, 30, and 60 min. The bioreduction reaction resulted in a color change in the mixture, resulting in a change from bright yellow (Au^3+^ ions) to reddish pink (characteristic of Au^0^ nanoparticles). The solution was centrifugated at a speed of 15,000 rpm for 10 min. The solid component was then dried in a 48 h drying procedure in an oven set at 85 °C. After that, the dehydrated fraction was crushed into a fine powder and stored in a sterile tube at +4 °C for characterization and future biological applications. 

### 3.2. Characterization of Biosynthesized AuNPs

After the color of the solution was changed, the material was examined using the Agilent CARY 60 (Agilent, Penang, Malaysia)device to evaluate the UV spectroscopy. The maximum absorption rate of CM-AuNPs has been measured as between 300 and 700 nm. D8 Discover computer-controlled X-ray diffraction (XRD) spectroscopy was used to analyze the X-ray fragmentation model and crystal size of biosynthesized AuNPs. The crystal nanoscale of CM-AuNPs was evaluated using X-ray diffraction (XRD) analysis. Measurements were carried out in the range of 20 to 80 degrees at 2θ. The Debye–Scherer equation (D = Kλ/(βcosθ)) was used to calculate the nanoscale of the crystal [64,65]. Fourier transformation infrared spectroscopy (FTIR) was used to investigate bioorganic functional groups found in the *C. moschata* peel and to contribute to the bioreduction process. The Perkin Elmer Spectrum 100 equipment was employed for this investigation, and the peaks between 500 and 3500 cm^−1^ were examined. The morphological appearance of the produced CM-AuNPs was determined using the FEI (Quanta 250 FEG) Scanning Electron Microscope (SEM). The element composition of pure gold or gold oxide particles was validated using data obtained from the energy-dispersive X-ray (RadB-DMAX II computer-controlled-EDX, edX, Cambridge, MA, USA). The morphology and size of the CM-AuNPs, as well as the monodispersed structure of the particles, were analyzed using a Transmission Electron Microscope (TEM) (Jeol Jem. 1010). TGA-DTA analysis (DTG-60H, Shimadzu, Kyoto, Japan) was used to evaluate thermogravimetric changes due to increased temperature in the material. This analysis was carried out at a temperature range of 0 °C to 800 °C, using a 10 °C/min heating rate and under a nitrogen atmosphere. The three-dimensional topographic structure of the CM-AuNPs was determined by the analysis of an Atomic Force Microscope (AFM/Park System XE-100, Park Systems Inc., Santa Clara, CA, USA). The zeta potential was measured using a Zetasizer Nano NS (Malvern, UK) at pH 2–12, which has a significant effect on stability. 

### 3.3. Analysis of Phenolic Compounds Using LC-ESI-MS/MS 

A solution was prepared by dissolving 100 mg of crude plant extract in 10 mL of methanol. The resulting solution was then diluted to a concentration of 2 mg/mL using a mixture of 50% methanol and high-purity water. The solution was subsequently passed through a 0.22 mm scale filter before being transferred to a bottle for LC-ESI/MS/MS (Liquid Chromatography and Mass Spectrometry) analysis. The Poroshell 120 EC-C18 (Agilent Technologies, Santa Clara, CA, USA) column was used to chromatographically isolate various components. For mass spectrometric detection, the Shimadzu LCMS-8040 model mass spectrometer was used. The LC-ESI-MS/MS data were analyzed using the LabSolutions software (Version 5.3) developed by Shimadzu (Kyoto, Japan). Phytochemicals were measured using Multiple Reaction Monitoring (MRM) technique. Research using mass spectrometry (MS) was carried out with specific experimental parameters [66].

### 3.4. Determination of Cytotoxic Effect by MTT Assay

The cytotoxicity analysis of biosynthesized AuNPs was carried out at the Cell Culture Laboratory of Dicle University Scientific Research Centre. The cytotoxic effect experiments used human cervical endothelial cells (HUVECs), human ovarian adenocarcinoma (Sk-Ov-3) cell series, human lung cancer (A549) cell series, and human colon adenocarcinoma (CaCo-2) cell cultures. Sk-Ov-3 cells were cultivated in the RPMI-1640 growth environment, while other cell types were cultured in the Dulbecco Modified Eagle environment (DMEM). The cell lines were grown in a humidified incubator with 95% atmospheric air and 5% CO_2_ at 37 °C. The cells controlled with the hemacytometer were then placed in 96-foot microplates and incubated throughout the night. The cell lines were cultivated in microplate wells containing CM-AuNPs at concentrations ranging from 6.25 to 100 μg/mL for 48 h at 37 °C in a CO_2_ incubator. After the incubation period, a solution of MTT (3-(4.5-dimetyltiazole-2-yl)-2,5-diphenyltetrazolium bromide) was added to the microplate pits and incubated for three hours [67]. The absorption at 540 nm was measured with the Thermo Multiscan GO. Using these absorbance values, the percentages of cell vitality were calculated according to the published formula [68].

### 3.5. Determination of Antipathogenic Effect by Microdilution Method

The inhibitory effect of CM-AuNPs on the growth of microorganisms was studied using Gram-negative (*Escherichia coli* ATCC 25922) and Gram-positive pathogen bacterial strains (*Staphylococcus aureus* ATSC 29213) and the fungus *Candida albicans*. The McFarland standard (0.5), Mueller Hinton, the RPMI feed medium, and antibiotics (fluconazole, vancomycin, and colistin) were commercially purchased from Sigma Aldrich for antipathogenic investigations. The minimum inhibitory concentration (MIC) was determined by microdilution to evaluate the antibacterial and antifungal properties of CM-AuNPs. After microdilution (100 μL beginning with the first well) of 96-well microplates, all plates were incubated for 24 h at 37 °C. The following day, the microplate wells were examined for signs of growth. The concentrations at which growth in the wells was inhibited were identified as MIC values. Fluconazole was used as a standard antibiotic for yeast, colistin for Gram-negative bacteria, and vancomycin for Gram-positive bacteria [69]. 

## 4. Conclusions

In this paper, the green synthesis of gold nanoparticles (AuNPs) was carried out using an aqueous extract of *C. moschata* peel using an environmentally friendly, low-cost, simple, and rapid method. UV, FTIR, TEM, AFM, SEM, EDX, TGA-DTA, XRD, and zeta potential measurements were performed to characterize the CM-AuNPs obtained as a result of the synthesis. The spherical morphology of AuNPs was shown by TEM examination with an average size of 18.10 nm. With the use of phytochemicals found in the aqueous extract of *C. moschata* peel as reducing agents, the biosynthesis process of CM-AuNPs was performed efficiently and was environmentally friendly, without any toxic or dangerous components. SEM and TEM images indicated the synthesis of high-stability spherically biogenic AuNPs. UV-vis, XRD, and EDX analyses confirmed the synthesis of AuNPs. The crystalline properties of AuNPs were determined using XRD analysis. Microscopic analyses have shown that AuNPs have predominantly spherical morphology, mostly with an average size of 18 to 24 nm. CM-AuNPs showed antimicrobial activity against Gram-positive *S. aureus*, Gram-negative *E. coli*, and *C. albicans* at concentrations of 0.004, 0.64, and 0.128 μg/mL. AuNPs showed strong antimicrobial activity at low concentrations. The cytotoxic activity of CM-AuNPs was evaluated using the MTT method. The concentration of 50 μg/mL of CM-AuNPs suppressed healthy cells by 50% and the vitality of cancer cell lines by 37–58%. It is thought that NPs can be used in many commercial products for biological and medical purposes, and this article emphasizes that CM-AuNPs have significant potential for highly effective antimicrobial and cancer treatment in the food industry and in medical applications. 

## Figures and Tables

**Table 1:** LC-MS/MS quantification results of *C. moschata* peel methanol extract.

No	Standards	RT	M.I. (*m*/*z*)	*R* ^2^	RSD	Linearity Range (µg/L)	LOD/LOQ (µg/L)	Recovery (%)		*C. moschata* Results (µg/mL)
								Interday	Intraday	
**1**	Quinic acid	3.0	190.8	0.996	0.69	0.1–5	25.7/33.3	1.0011	1.0083	ND
**2**	Fumaric aid	3.9	115.2	0.995	1.05	1–50	135.7/167.9	0.9963	1.0016	25.24
**3**	Aconitic acid	4.0	172.8	0.971	2.07	0.1–5	16.4/31.4	0.9968	1.0068	ND
**4**	Gallic acid	4.4	168.8	0.999	1.60	0.1–5	13.2/17.0	1.0010	0.9947	ND
**5**	Epigallocatechin	6.7	304.8	0.998	1.22	1–50	237.5/265.9	0.9969	1.0040	ND
**6**	Protocatechuic acid	6.8	152.8	0.957	1.43	0.1–5	21.9/38.6	0.9972	1.0055	0.094
**7**	Catechin	7.4	288.8	0.999	2.14	0.2–10	55.0/78.0	1.0024	1.0045	ND
**8**	Gentisic acid	8.3	152.8	0.997	1.81	0.1–5	18.5/28.2	0.9963	1.0077	ND
**9**	Protocatechuic aldehyde	8.5	137.2	0.996	2.08	0.1–5	15.4/22.2	1.0002	0.9988	0.622
**10**	Chlorogenic acid	8.4	353.0	0.995	2.15	0.1–5	13.1/17.6	1.0000	1.0023	ND
**11**	Tannic acid	9.2	182.8	0.999	2.40	0.05–2.5	15.3/22.7	0.9970	0.9950	ND
**12**	Epigallocatechin gallate	9.4	457.0	0.999	1.30	0.2–10	61.0/86.0	0.9981	1.0079	ND
**13**	1,5-dicaffeoylquinic acid	9.8	515.0	0.999	2.42	0.1–5	5.8/9.4	0.9983	0.9997	ND
**14**	4-OH Benzoic acid	10.5	137,2	0.999	1.24	0.2–10	68.4/88.1	1.0032	1.0068	ND
**15**	Epicatechin	11.6	289.0	0.996	1.47	1–50	139.6/161.6	1.0013	1.0012	ND
**16**	Vanillic acid	11.8	166.8	0.999	1.92	1–50	141.9/164.9	1.0022	0.9998	ND
**17**	Caffeic acid	12.1	179.0	0.999	1.11	0.05–2.5	7.7/9.5	1.0015	1.0042	ND
**18**	Syringic acid	12.6	196.8	0.998	1.18	1–50	82.3/104.5	1.0006	1.0072	ND
**19**	Vanillin	13.9	153.1	0.996	1.10	0.1–5	24.5/30.4	1.0009	0.9967	0.211
**20**	Syringic aldehyde	14.6	181.0	0.999	2.51	0.4–20	19.7/28.0	1.0001	0.9964	ND
**21**	Daidzin	15.2	417.1	0.996	2.25	0.05–2.5	7.0/9.5	0.9955	1.0017	ND
**22**	Epicatechin gallate	15.5	441.0	0.997	1.63	0.1–5	19.5/28.5	0.9984	0.9946	ND
**23**	Piceid	17.2	391.0	0.999	1.94	0.05–2.5	13.8/17.8	1.0042	0.9979	ND
**24**	p-Coumaric acid	17.8	163.0	0.999	1.92	0.1–5	25.9/34.9	1.0049	1.0001	0.62
**25**	Ferulic acid-D3-IS	18.8	196.2	N.A.	N.A.	N.A.	N.A.	N.A.	N.A.	ND
**26**	Ferulic acid	18.8	192.8	0.999	1.44	1–50	11.8/15.6	0.9951	0.9976	ND
**27**	Sinapic acid	18.9	222.8	0.999	1.45	0.2–10	65.2/82.3	1.0031	1.0037	ND
**28**	Coumarin	20.9	146.9	0.999	2.11	0.05–2.5	214.2/247.3	0.9950	0.9958	ND
**29**	Salicylic acid	21.8	137.2	0.999	1.48	0.05–2.5	6.0/8.3	0.9950	0.9998	ND
**30**	Cynaroside	23.7	447.0	0.997	1.56	0.05–2.5	12.1/16.0	1.0072	1.0002	ND
**31**	Miquelianin	24.1	477.0	0.999	1.31	0.1–5	10.6/14.7	0.9934	0.9965	ND
**33**	Rutin	25.6	608.9	0.999	1.38	0.1–5	15.7/22.7	0.9977	1.0033	ND
**34**	Isoquercitrin	25.6	463.0	0.998	2.13	0.1–5	8.7/13.5	1.0057	0.9963	ND
**35**	Hesperidin	25.8	611.2	0.999	1.84	0.1–5	19.0/26.0	0.9967	1.0043	ND
**36**	o-Coumaric acid	26.1	162.8	0.999	2.11	0.1–5	31.8/40.4	1.0044	0.9986	ND
**37**	Genistin	26.3	431.0	0.991	2.01	0.1–5	14.9/21.7	1.0062	1.0047	ND
**38**	Rosmarinic acid	26.6	359.0	0.999	1.24	0.1–5	16.2/21.2	1.0056	1.0002	ND
**39**	Ellagic acid	27.6	301.0	0.999	1.57	0.4–20	56.9/71.0	1.0005	1.0048	ND
**40**	Cosmosiin	28.2	431.0	0.998	1.65	0.1–5	6.3/9.2	0.9940	0.9973	ND
**41**	Quercitrin	29.8	447.0	0.999	2.24	0.1–5	4.8/6.4	0.9960	0.9978	ND
**42**	Astragalin	30.4	447.0	0.999	2.08	0.1–5	6.6/8.2	0.9968	0.9957	ND
**43**	Nicotiflorin	30.6	592.9	0.999	1.48	0.05–2.5	11.9/16.7	0.9954	1.0044	ND
**44**	Fisetin	30.6	285.0	0.999	1.75	0.1–5	10.1/12.7	0.9980	1.0042	ND
**45**	Daidzein	34.0	253.0	0.999	2.18	0.1–5	9.8/11.6	0.9926	0.9963	ND
**46**	Quercetin-D3-IS	35.6	304.0	N.A.	N.A.	N.A.	N.A.	N.A.	N.A.	ND
**47**	Quercetin	35.7	301.0	0.999	1.89	0.1–5	15.5/19.0	0.9967	0.9971	ND
**48**	Naringenin	35.9	270.9	0.999	2.34	0.1–5	2.6/3.9	1.0062	1.0020	0.357
**49**	Hesperetin	36.7	301.0	0.999	2.47	0.1–5	7.1/9.1	0.9998	0.9963	ND
**50**	Luteolin	36.7	284.8	0.999	1.67	0.05–2.5	2.6/4.1	0.9952	1.0029	ND
**51**	Genistein	36.9	269.0	0.999	1.48	0.05–2.5	3.7/5.3	1.0069	1.0012	ND
**52**	Kaempferol	37.9	285.0	0.999	1.49	0.05–2.5	10.2/15.4	0.9992	0.9990	0.011
**53**	Apigenin	38.2	268.8	0.998	1.17	0.05–2.5	1.3/2.0	0.9985	1.0003	0.01
**54**	Amentoflavone	39.7	537.0	0.992	1.35	0.05–2.5	2.8/5.1	0.9991	1.0044	ND
**55**	Chrysin	40.5	252.8	0.999	1.46	0.05–2.5	1.5/2.8	0.9922	1.0050	ND
**56**	Acacetin	40.7	283.0	0.997	1.67	0.02–1	1.5/2.5	0.9949	1.0011	0.012

RT: retention time; M.I. (*m*/*z*): molecular ions of the standard analytes (*m*/*z* ratio); R^2^: coefficient of determination; RSD: relative standard deviation; LOD/LOQ (µg/L): limit of detection/quantification; N.A: no answer; ND: not determined.

**Table 2:** The evaluation of the viability of CM-AuNPs by their interaction on cell lines.

Cell Lines	Control	6.25 μg/mL	12.5 μg/mL	25 μg/mL	50 μg/mL	100 μg/mL	IC_50_
**HUVEC**	100	59.85	61.75	52.11	49.31	35.04	44.6747
**Sk-Ov-3**	100	86.76	84.55	77.17	42.76	64.98	114.0851
**A549**	100	70.96	75.84	74.53	47.19	63.47	144.2599
**CaCo-2**	100	73.54	72.59	64.95	63.95	73.27	1871.2831

**Table 3:** Comparative analysis of the inhibiting concentrations of green-synthesized AuNPs on different cancer cell lines, based on the % of viability.

Biologic Material	Tested Cell	Shape	Size (nm)	Effective Concentration (μg/mL)	Reference
*Trichosanthes kirilowii*	HCT-116	Spherical	50	5.5	[17]
*Lagenaria siceraria*	A549	Spherical	40–50	100	[18]
*Hygrophila spinosa*	Sk-Ov-3	Spherical	68.44	200	[55]
*Hubertia ambavilla*	NHDF	Spherical	97.7	25	[56]
*Caulerpa racemosa*	H460	Spherical	18–45	25	[57]
*Gundelia tournefortii*	CaCo-2	Spherical	23.53	25	[58]

**Table 4:** The concentrations of AuNPs that effectively inhibited the growth of microorganisms in various green synthesis investigations utilizing biologically originated materials.

AuNPs MIC Values in µg/mL				
Biosynthesis Source	Gram-Positive *S. aureus*	Gram-Negative *E. coli*	*C. albicans*	Reference
*Jatropha integerrima*	10	2.5	-	[14]
*Cucurbita pepo.*	800	400	*-*	[16]
*Benincasa hispida*	26.9	21.6	-	[25]
*Allium ampeloprasum*	0.0612	0.50	0.125	[39]
*Gundelia tournefortii*	0.25	1.0	0.5	[62]
*Crataegus monogyna*	0.056	0.50	0.112	[63]

**Table 5:** MIC values of synthesized CM-AuNPs, HAuCI_4_, and antibiotics (µg/mL).

Microorganisms	CM-AuNPs	HAuCI_4_	Standard Antibiotics *
Gram (+) *S. aureus* ATCC 29213	0.004	0.25	1.00
Gram (−) *E. coli* ATCC 25922	0.64	1.00	2.00
*C. albicans*	0.128	0.50	1.00

* Antibiotics: Colistin (Gram-positive bacteria), vancomycin (Gram-negative bacteria), and fluconazole (*C. albicans*).

## Data Availability

All data used to support the findings of this study are included in the article.

## References

[1] Lloyd-Hughes H., Shiatis A.E., Pabari A., Mosahebi A., Seifalian A. (2015). Current and future nanotechnology applications in the management of melanoma: A review. J. Nanomed. Nanotechnol..

[2] Abou El-Nour K.M., Eftaiha A.A., Al-Warthan A., Ammar R.A. (2010). Synthesis and applications of silver nanoparticles. Arab. J. Chem..

[3] Rauf A., Ahmad T., Khan A.M., Uddin G., Ahmad B., Al-Harrasi A. (2021). Green synthesis and biomedicinal applications of silver and gold nanoparticles functionalized with methanolic extract of *Mentha longifolia*. Artif. Cells Nanomed. Biotechnol..

[4] Vijayaraghavan K., Ashokkumar T. (2017). Plant-mediated biosynthesis of metallic nanoparticles: A review of literature, factors affecting synthesis, characterization techniques, and applications. J. Environ. Chem. Eng..

[5] Mout R., Moyano D.F., Rana S., Rotello V.M. (2012). Surface functionalization of nanoparticles for nanomedicine. Chem. Soc. Rev..

[6] Ding X., Li D., Jiang J. (2020). Gold-based inorganic nanohybrids for nanomedicine applications. Theranostics.

[7] Siddiq A.M., Thangam R., Madhan B., Alam M.S. (2019). Green (gemini) surfactant mediated gold nanoparticles green synthesis: Effect on triple negative breast cancer cells. Nano-Struct. Nano-Objects.

[8] Nagajyothi P.C., Lee K.D. (2011). Synthesis of plant-mediated silver nanoparticles using *Dioscorea batatas* rhizome extract and evaluation of their antimicrobial activities. J. Nanomater..

[9] Mustapha T., Misni N., Ithnin N.R., Daskum A.M., Unyah N.Z. (2022). A review on plants and microorganisms mediated synthesis of silver nanoparticles, role of plants metabolites and applications. Int. J. Environ. Res. Public Health.

[10] Soto K.M., Mendoza S., López-Romero J.M., Gasca-Tirado J.R., Manzano-Ramírez A. (2021). Gold nanoparticles: Synthesis, application in colon cancer therapy and new approaches-review. Green Chem. Lett. Rev..

[11] Prabhu S., Poulose E.K. (2012). Silver nanoparticles: Mechanism of antimicrobial action, synthesis, medical applications, and toxicity effects. Int. Nano Lett..

[12] Rolnik A., Olas B. (2020). Vegetables from the Cucurbitaceae family and their products: Positive effect on human health. Nutrition.

[13] Adams G.G., Imran S., Wang S., Mohammad A., Kok S., Gray D.A., Channell G.A., Morris G.A., Harding S.E. (2011). The hypoglycaemic effect of pumpkins as anti-diabetic and functional medicines. Food Res. Int..

[14] Suriyakala G., Sathiyaraj S., Babujanarthanam R., Alarjani K.M., Hussein D.S., Rasheed R.A., Kanimozhi K. (2022). Green synthesis of gold nanoparticles using *Jatropha integerrima* Jacq. flower extract and their antibacterial activity. J. King Saud Univ. Sci..

[15] Al Saqr A., Khafagy E.-S., Alalaiwe A., Aldawsari M.F., Alshahrani S.M., Anwer M.K., Khan S., Lila A.S.A., Arab H.H., Hegazy W.A.H. (2021). Synthesis of gold nanoparticles by using green machinery: Characterization and in vitro toxicity. Nanomaterials.

[16] Chandran K., Song S., Yun S.I. (2019). Effect of size and shape controlled biogenic synthesis of gold nanoparticles and their mode of interactions against foodborne bacterial pathogens. Arab. J. Chem..

[17] Han X., Jiang X., Guo L., Wang Y., Veeraraghavan V.P., Krishna Mohan S., Wang Z., Cao D. (2019). Anticarcinogenic potential of gold nanoparticles synthesized from *Trichosanthes kirilowii* in colon cancer cells through the induction of apoptotic pathway. Artif. Cells Nanomed. Biotechnol..

[18] Kumar V., Hussain P.R., Chatterjee S., Variyar P.S. (2015). Evaluation of in vitro antioxidant activity and characterization of phenolic compounds of bottle gourd towards the green synthesis of gold nanoparticles and its bio-efficacy. Int J Food Nutr Saf.

[19] Al-Ardi M.H. (2020). The uses of gold nanoparticles and *Citrullus colocynthis* L. nanoparticles against *Giardia lamblia* in vivo. Clin. Epidemiol. Glob. Health.

[20] Duong T.G., Phan T.L., Nguyen T.L.H., Chau T.P., Doan V.D. (2021). Effective reduction of nitrophenols and colorimetric detection of Pb (ii) ions by *Siraitia grosvenorii* fruit extract capped gold nanoparticles. RSC Adv..

[21] Ahmad W., Kalra D. (2020). Green synthesis, characterization and antimicrobial activities of ZnO nanoparticles using *Euphorbia hirta* leaf extract. J. King Saud Univ. Sci..

[22] Dos Santos W.N.L., da Silva Sauthier M.C., dos Santos A.M.P., de Andrade Santana D., Azevedo R.S.A., da Cruz Caldas J. (2017). Simultaneous determination of 13 phenolic bioactive compounds in guava (*Psidium guajava* L.) by HPLC-PAD with evaluation using PCA and Neural Network Analysis (NNA). Microchem. J..

[23] Kakkar S., Bais S. (2014). A review on protocatechuic acid and its pharmacological potential. Int. Sch. Res. Not..

[24] Enneb S., Drine S., Bagues M., Triki T., Boussora F., Guasmi F., Nagaz K., Ferchichi A. (2020). Phytochemical profiles and nutritional composition of squash (*Cucurbita moschata* D.) from Tunisia. South Afr. J. Bot..

[25] Trodtfeld F., Tölke T., Wiegand C. (2022). Antimicrobial Functionalization of Prolamine–Silica Hybrid Coatings with Fumaric Acid for Food Packaging Materials and Their Biocompatibility. Antibiotics.

[26] Amini S.M., Akbari A. (2019). Metal nanoparticles synthesis through natural phenolic acids. IET Nanobiotechnol..

[27] Özçelik B., Kartal M., Orhan I. (2011). Cytotoxicity, antiviral, and antimicrobial activities of alkaloids, flavonoids, and phenolic acids. Pharm. Biol..

[28] Singh G., Pahari A.K., Leo V.V., Mishra V.K., Subbarayan S., Singh B.P., Kumar B., Kumar S., Gupta V.K., Nachimuthu S.K. (2016). Evaluation of phenolic content variability along with antioxidant, antimicrobial, and cytotoxic potential of selected traditional medicinal plants from India. Front. Plant Sci..

[29] Huang X., Devi S., Bordiga M., Brennan C.S., Xu B. (2023). Phenolic compounds mediated biosynthesis of gold nanoparticles and evaluation of their bioactivities: A review. Int. J. Food Sci. Technol..

[30] Liu W.H., Hsu C.C., Yin M.C. (2008). In vitro anti-*Helicobacter pylori* activity of diallyl sulfides and protocatechuic acid. Phytother. Res. Int. J. Devoted Pharmacol. Toxicol. Eval. Nat. Prod. Deriv..

[31] Zhou L., Zuo Z., Chow M.S.S. (2005). Danshen: An overview of its chemistry, pharmacology, pharmacokinetics, and clinical use. J. Clin. Pharmacol..

[32] Dubey S.P., Lahtinen M., Sillanpää M. (2010). Green synthesis and characterizations of silver and gold nanoparticles using leaf extract of *Rosa rugosa*. Colloids Surf. A Physicochem. Eng. Asp..

[33] Gonnelli C., Cacioppo F., Giordano C., Capozzoli L., Salvatici C., Salvatici M.C., Colzi I., Del Bubba M., Ancillotti C., Ristori S. (2015). *Cucurbita pepo* L. extracts as a versatile hydrotropic source for the synthesis of gold nanoparticles with different shapes. Green Chem. Lett. Rev..

[34] Iyer R.I., Panda T. (2018). Biosynthesis of gold and silver nanoparticles using extracts of callus cultures of pumpkin (*Cucurbita maxima*). J. Nanosci. Nanotechnol..

[35] Patra J.K., Kwon Y., Baek K.H. (2016). Green biosynthesis of gold nanoparticles by onion peel extract: Synthesis, characterization, and biological activities. Adv. Powder Technol..

[36] Baran A. (2023). Inhibitory effects of gold nanoparticles biosynthesized by redox reaction *using Rheum ribes* lam fruit peels on pathogen strains and cancer cells. Part Sci. Technol..

[37] Babu B., Palanisamy S., Vinosha M., Anjali R., Kumar P., Pandi B., Tabarsa M., You S.G., Prabhu N.M. (2020). Bioengineered gold nanoparticles from marine seaweed *Acanthophora spicifera* for pharmaceutical uses: Antioxidant, antibacterial, and anticancer activities. Bioprocess Biosyst. Eng..

[38] Baran M.F., Acay H., Keskin C. (2020). Determination of antimicrobial and toxic metal removal activities of plant-based synthesized (*Capsicum annuum* L. Leaves), eco-friendly, gold nanomaterials. Glob. Chall..

[39] Hatipoğlu A. (2021). Rapid green synthesis of gold nanoparticles: Synthesis, characterization and antimicrobial activities. Prog. Nutr.

[40] Shah M., Fawcett D., Sharma S., Tripathy S.K., Poinern G.E.J. (2015). Green synthesis of metallic nanoparticles via biological entities. Materials.

[41] Donga S., Bhadu G.R., Chanda S. (2020). Antimicrobial, antioxidant, and anticancer activities of gold nanoparticles green synthesized using *Mangifera indica* seed aqueous extract. Artif. Cells Nanomed. Biotechnol..

[42] Sunderam V., Thiyagarajan D., Lawrence A.V., Mohammed S.S.S., Selvaraj A. (2019). In-vitro antimicrobial and anticancer properties of green synthesized gold nanoparticles using *Anacardium occidentale* leaves extract. Saudi J. Biol. Sci..

[43] Geethalakshmi R., Ashokkumar T., Tamilselvan S., Govindaraju K., Sadiq M., Singaravelu G. (2013). Green synthesis of gold nanoparticles and their anticancer activity. Cancer Nanotechnol..

[44] Manikandakrishnan M., Palanisamy S., Vinosha M., Kalanjiaraja B., Mohandoss S., Manikandan R., Tabarsa M., You S., Prabhu N.M. (2019). Facile green route synthesis of gold nanoparticles using *Caulerpa racemosa* for biomedical applications. J. Drug Deliv. Sci. Technol..

[45] Umamaheswari C., Lakshmanan A., Nagarajan N.S. (2018). Green synthesis, characterization and catalytic degradation studies of gold nanoparticles against congo red and methyl orange. J. Photochem. Photobiol. B Biol..

[46] Pandey S., Oza G., Mewada A., Sharon M. (2012). Green synthesis of highly stable gold nanoparticles using *Momordica charantia* as nano fabricator. Arch. Appl. Sci. Res..

[47] Baran M.F., Saydut A. (2019). Altın nanomalzeme sentezi ve karekterizasyonu. Dicle Üniversitesi Mühendislik Fakültesi Mühendislik Derg..

[48] Arunachalam R., Dhanasingh S., Kalimuthu B., Uthirappan M., Rose C., Mandal A.B. (2012). Phytosynthesis of silver nanoparticles using *Coccinia grandis* leaf extract and its application in the photocatalytic degradation. Colloids Surf. B Biointerfaces.

[49] Lee K.X., Shameli K., Yew Y.P., Teow S.Y., Jahangirian H., Rafiee-Moghaddam R., Webster T.J. (2020). Recent developments in the facile bio-synthesis of gold nanoparticles (AuNPs) and their biomedical applications. Int. J. Nanomed..

[50] Hemlata P.R.M., Singh A.P., Tejavath K.K. (2020). Biosynthesis of silver nanoparticles using cucumis prophetarum aqueous leaf extract and their antibacterial and antiproliferative activity against cancer cell lines. ACS Omega.

[51] Paramasivam V., Paulpandian P., Venkatachalam K., Hussain S., Kangal A., Al Farraj D.A., Elshikh M.S., Balaji P. (2023). Cytotoxicity and Antimicrobial efficiency of gold (Au) nanoparticles formulated by green approach using *Andrographis paniculata* leaf extract. J. King Saud Univ. Sci..

[52] Mani M., Harikrishnan R., Purushothaman P., Pavithra S., Rajkumar P., Kumaresan S., Al Farraj D.A., Elshikh M.S., Balasubramanian B., Kaviyarasu K. (2021). Systematic green synthesis of silver oxide nanoparticles for antimicrobial activity. Environ. Res..

[53] Pertiwi R.D., Suwaldı E.P.S., Martien R. (2019). Bıo-Nanopartıcles: Green Synthesıs of Gold Nanopartıcles And Assessment of Bıologıcal Evaluatıon. Int. J. App. Pharm..

[54] Sukirtha R., Priyanka K.M., Antony J.J., Kamalakkannan S., Ramar T., Palani G. (2011). Cytotoxic effect of green synthesized silver nanoparticles using *Melia azedarach* against in vitro HeLa cell lines and Lymphoma mice model. Process Biochem..

[55] Satpathy S., Patra A., Ahirwar B., Hussain M.D. (2020). Process optimization for green synthesis of gold nanoparticles mediated by extract of *Hygrophila spinosa* T. Anders and their biological applications. Phys. E Low-Dimens. Syst. Nanostruct..

[56] Haddada M.B., Gerometta E., Chawech R., Sorres J., Bialecki A., Pesnel S., Spadavecchia J., Morel A.L. (2020). Assessment of antioxidant and dermoprotective activities of gold nanoparticles as safe cosmetic ingredient. Colloids Surf. B Biointerfaces.

[57] Pitchai P., Subramani P., Selvarajan R., Sankar R., Vilwanathan R., Sibanda T. (2022). Green synthesis of gold nanoparticles (AuNPs) using *Caulerpa racemosa* and evaluation of its antibacterial and cytotoxic activity against human lung cancer cell line. Arab J. Basic Appl. Sci..

[58] Keskin C., Baran A., Baran M.F., Hatipoğlu A., Adican M.T., Atalar M.N., Eftekhari A. (2020). Green synthesis, characterization of gold nanomaterials using *Gundelia tournefortii* leaf extract, and determination of their nanomedicine (antibacterial, antifungal, and cytotoxic) potential. J. Nanomater..

[59] Umadevi M., Rani T., Balakrishnan T., Ramanibai R. (2011). Antimicrobial activity of silver nanoparticles prepared under an ultrasonic field. Int. J. Pharm. Sci. Nanotechnol..

[60] Cui Y., Zhao Y., Tian Y., Zhang W., Lü X., Jiang X. (2012). The molecular mechanism of action of bactericidal gold nanoparticles on *Escherichia coli*. Biomater..

[61] Maillard A.P.F., Dalmasso P.R., de Mishima B.A.L., Hollmann A. (2018). Interaction of green silver nanoparticles with model membranes: Possible role in the antibacterial activity. Colloids Surf. B Biointerfaces.

[62] Rashidipour M., Heydari R. (2014). Biosynthesis of silver nanoparticles using extract of olive leaf: Synthesis and in vitro cytotoxic effect on MCF-7 cells. J. Nanostruct. Chem..

[63] Baran A., Hatipoğlu A., Baran M.F., Aktepe N. (2021). Synthesis of gold nanoparticles from hawthorn (*Crataegus monogyna*) fruit extract and evaluation of antimicrobial activities. Int. Conf. Des..

[64] Keskin C., Atalar M.N., Baran M.F., Baran A. (2021). Environmentally friendly rapid synthesis of gold nanoparticles from *Artemisia absinthium* plant extract and application of antimicrobial activities. J. Inst. Sci. Technol..

[65] Aktepe N., Baran A. (2021). Biosynthesis of AgNPs by extract from waste leaves of *Citrullus lanatus* sp.(watermelon); characterization, antibacterial and antifungal effects. Prog. Nutr..

[66] Yilmaz M.A. (2020). Simultaneous quantitative screening of 53 phytochemicals in 33 species of medicinal and aromatic plants: A detailed, robust and comprehensive LC–MS/MS method validation. Ind. Crops Prod..

[67] Elshikh M., Ahmed S., Funston S., Dunlop P., McGaw M., Marchant R., Banat I.M. (2016). Resazurin-based 96-well plate microdilution method for the determination of the minimum inhibitory concentration of biosurfactants. Biotechnol. Lett..

[68] Remya R.R., Rajasree S.R., Aranganathan L., Suman T.Y. (2015). An investigation on cytotoxic effect of bioactive AgNPs synthesized using *Cassia fistula* flower extract on breast cancer cell MCF-7. Biotechnol. Rep..

[69] Kahlmeter G., Brown D.F.J., Goldstein F.W., MacGowan A.P., Mouton J.W., Odenholt I., Rodloff A., Soussy C.-J., Steinbakk M., Soriano F. (2006). European Committee on Antimicrobial Susceptibility Testing (EUCAST) technical notes on antimicrobial susceptibility testing. Clin. Microbiol. Infect..

